# Impact of the Medical Faculty on Study Success in Freiburg: Results from Graduate Surveys

**DOI:** 10.3205/zma000986

**Published:** 2015-10-15

**Authors:** Silke Biller, Martin Boeker, Götz Fabry, Marianne Giesler

**Affiliations:** 1 Universität Basel, Medizinische Fakultät, Studiendekanat, Basel, Schweiz; 2 Universitätsklinikum Freiburg, Department für Medizinische Biometrie und Medizinische Informatik, Freiburg, Deutschland; 3 Albert-Ludwigs-Universität Freiburg, Bereich für Medizinische Psychologie und Medizinische Soziologie, Freiburg, Deutschland; 4 Universität Freiburg, Medizinische Fakultät, Studiendekanat, Freiburg, Deutschland; 5 Kompetenzzentrum Lehrevaluation in der Medizin Baden-Württemberg, Sitz Freiburg, Deutschland

**Keywords:** Graduate survey, teaching evaluation, quality improvement, study success

## Abstract

**Aim: **Using the data from graduate surveys, this study aims to analyze which factors related to teaching and learning at the Freiburg Faculty of Medicine can influence study success.

**Background:** Study success and the factors influencing it have long been the subject of investigation, with study success being measured in terms of easily quantifiable indicators (final grades, student satisfaction, etc.). In recent years, it has also frequently been assessed in terms of graduate competency levels. Graduate surveys are considered suitable instruments for measuring these dimensions of study success.

**Method:** Data from three Freiburg graduate surveys conducted one and a half years after graduation were drawn upon for the analysis.

Study success was operationalized using four indicators: results on the written section of the M2 exam, self-assessment of medical expertise and scientific expertise, and student satisfaction. Using multiple regression analyses, the predictive power was calculated for selected variables, also measured by the graduate surveys, for the different study success indicators.

**Results:** It was possible to identify models that contribute slightly or moderately to the prediction of study success. The score earned on the university entrance qualification demonstrated itself to be the strongest predictor for forecasting the M2 written exam: R^2^ is between 0.08 and 0.22 for the three surveys. Different variables specific to degree program structure and teaching are helpful for predicting medical expertise (R^2^=0.04-0.32) and student satisfaction (R^2^=0.12-0.35). The two variables, *structure and curricular sequencing of the degree program* and *combination of theory and practice*, show themselves to be significant, sample-invariant predictors (β-weight_Structure_=0.21-0.58, β-weight_Combination_=0.27-0.56). For scientific expertise, no sample-independent predictors could be determined.

**Conclusion:** Factors describing teaching hardly provide any assistance when predicting the written M2 exam score, which makes sense to the extent that teaching goes far beyond the heavily knowledge-based content of the written M2 exam. The lack of predictability for scientific expertise is most likely explained in that these have been only rarely included in the curriculum and often inexplicitly so. The variable *combination of theory and practice* appears to be significant for imparting medical expertise and the development of student satisfaction. The extent to which these relationships are practically relevant needs to be explored in further studies.

A specific limitation is that the measurement of expertise and skill is based solely on self-assessments.

## Zusammenfassung

**Zielsetzung: **Zielsetzung der Arbeit ist es, mit Hilfe der Daten von Absolventenbefragungen zu untersuchen, welche lehr- und lernbezogenen Bedingungen der Medizinischen Fakultät Freiburg den Studienerfolg beeinflussen können.

**Hintergründe: **Der Studienerfolg und ihn beeinflussende Faktoren werden seit langem untersucht, wobei der Studienerfolg an leicht quantifizierbaren Indikatoren gemessen wird (z.B. Abschlussnoten, Studienzufriedenheit), in den letzten Jahren auch häufig anhand des Kompetenzniveaus der Absolventen. Absolventenbefragungen gelten als geeignete Instrumente um, diese Dimensionen des Studienerfolgs zu erfassen.

**Methodik: **Zur Analyse wurden die Daten von drei Freiburger Absolventenbefragungen herangezogen, die jeweils 1,5 Jahre nach Studienabschluss erfolgten.

Studienerfolg wurde über vier Indikatoren operationalisiert: Ergebnisse des M2 Examens (M2-schriftlich), selbsteingeschätzte Fach- und Wissenschaftskompetenz und Studienzufriedenheit. Mittels multipler Regressionsanalysen wurde die Vorhersagekraft ausgewählter, ebenfalls mit dem Absolventenfragebogen erhobener Variablen für die verschiedenen Studienerfolgsindikatoren berechnet.

**Ergebnisse: **Es konnten Modelle identifiziert werden, die wenig bis mäßig zur Vorhersage des Studienerfolgs beitragen. Die Note der Hochschulzugangsberechtigung erweist sich als stärkster Prädiktor zur Vorhersage von M2-schriftlich: R^2^ liegt hier zwischen 0.08 und 0.22 für die drei Befragungen. Zur Vorhersage der Fachkompetenzeinschätzung (R^2^=0.04-0.32) und der Studienzufriedenheit (R^2^=0.12-0.35) tragen verschiedene Variablen des Studienaufbaus und des Lehrprozesses bei. Die Variablen *Aufbau und Struktur des Studiums* und *Verknüpfung von Theorie und Praxis* erweisen sich als signifikante, stichprobeninvariante Prädiktoren (β-Gewicht_Aufbau_=0.21-0.58, β-Gewicht_Verknüpf_=0.27-0.56,). Für Wissenschaftskompetenz konnten keine stichprobenunabhängige Prädiktoren ermittelt werden.

**Schlussfolgerungen:** Faktoren, die die Lehre beschreiben, leisten kaum einen Beitrag zur Vorhersage der schriftlichen M2-Note, was insofern nachvollziehbar ist, als die Lehre weit über den stark wissensorientierten Inhalt des M2-schriftlich hinausgeht. Die mangelnde Vorhersagbarkeit der Wissenschaftskompetenz ist am ehesten damit erklärbar, dass diese Kompetenz bisher nur wenig und häufig nicht expliziter Bestandteil des Curriculums ist. Die Variable *Verknüpfung von Theorie und Praxis* scheint für die Ausbildung der Fachkompetenz und die Ausprägung der Studienzufriedenheit bedeutsam zu sein. Inwieweit diese Beziehungen praktisch relevant sind, soll in weiteren Studien geklärt werden.

Limitierend ist insbesondere anzumerken, dass die Kompetenzerfassung ausschließlich auf Selbsteinschätzungen beruht.

## Introduction

For a long time now, research in education has focused on identifying which factors (personal, institutional, etc.) influence study success. The construct study success has been given varying definitions. Often, easily quantifiable academic achievements are drawn upon as indicators, such as length of study, overall grade, or the successful completion of a particular degree program. However, in recent years the competencies acquired during the course of study have increasingly gained importance. In particular, the professional preparation of graduates is to be improved as a result of this increasing focus on competency. A prototype for this development in medicine, the CanMEDS model [1], was originally designed for advanced and continuing medical education. Meanwhile, it is also used in a modified form as a guiding framework for medical degree programs and the National Competency-based Catalogue of Learning Objectives in Undergraduate Medical Education (NKLM) [2]. Within the scope of quality assurance in education, statements on outcomes are increasingly expected of universities [http://www.akkreditierungsrat.de/fileadmin/Seiteninhalte/AR/Beschluesse/AR_Regeln_Studiengaenge_aktuell.pdf, most recently viewed on 28 Jan. 2014]. To answer part of this growing need for evaluations, medical schools in Germany have responded with a series of graduate surveys [3]. Graduate surveys allow the assertion of concrete statements about the attainment of educational objectives in respect to outcomes, such as study success and the professional qualification of the students [4], [5].

**Figure 1:**
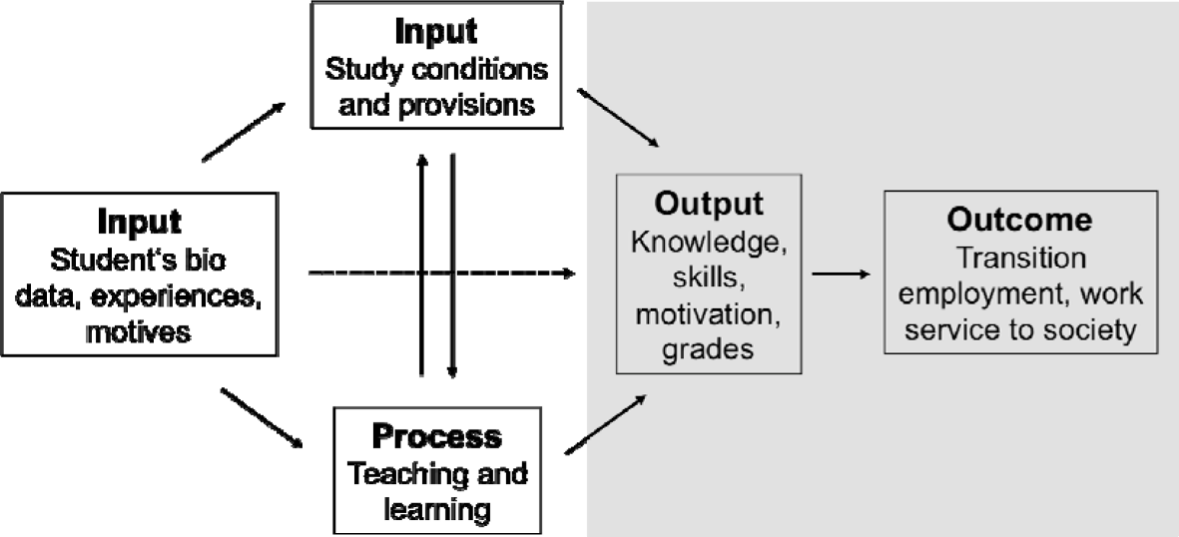
Analytical model for graduate surveys according to Schomburg 2003.

Using Schomburg’s model of analysis for graduate surveys [6] (see Figure 1 (Fig. 1)), it is possible to pose questions about connections between indicators for study success and aspects such as personal background, individual assessments of study conditions, and the teaching/learning processes. Influential factors within the context of an individual background are assumed in the model. This can act to substantially moderate the effect the independent variables of study program, study conditions and teaching/learning processes have on the dependent variables. As a result, all three conditions have direct or indirect influence on the primary outcomes of university study (knowledge, competencies, scores, motivation), which according to Teichler [7] are seen as the direct result of the universities. The primary outcome, in turn, influences secondary and tertiary results, which encompass the professional career (transition to career, application and use of competencies, contribution to society) [3].

The primary results have particular meaning for the development of quality at universities, since these are connected most closely with the work of universities.

This study defined the following graduate survey parameters as indicators of study success to see which teaching and learning conditions at the university are important for them:

1. Written section of the second part of the medical examination (written M2 exam): The final scores are regularly drawn upon in the literature as indicators of study success. In medical degree programs these exams are administered as multiple-choice tests and possess a high degree of objectivity and reliability.
2. Competencies: Work on the National Catalogue of Competency-based Learning Objectives in Undergraduate Medical Education (NKLM) has been underway since 2009 [2], it was passed with agreement at the annual meeting of the Association of Medical Faculties in Germany (MFT) in 2015 [http://www.nklm.de]. Based on the CanMEDS roles [1], seven physician competencies have been defined. At present, not all of the competency roles envisioned by the NKLM have been explicitly integrated into the curriculum. Hence, this study only defines as indicators those roles for which it could be assumed that they were already part of the curriculum at the time the graduate surveys were conducted. These roles include the *Medical Expert and Scholar*, the latter currently the subject of many discussions about the *Physician as Researcher* [8].
3. Student satisfaction: The importance of student satisfaction as an indicator for study success is approached in differing manners. In the US literature [9], satisfaction with a specific study program is viewed as an important and complex construct that, for instance, makes statements possible about students’ sense of belonging or loyalty to a particular institution. It correlates strongly with student commitment and is considered to be heavily dependent on the university environment. In contrast, the German literature is more critical in discussing study satisfaction in reference to study success. Rindermann [10] points out that student satisfaction compares individual needs with objective study program quality making the term unclear in its meaning [11].

**Figure 2:**
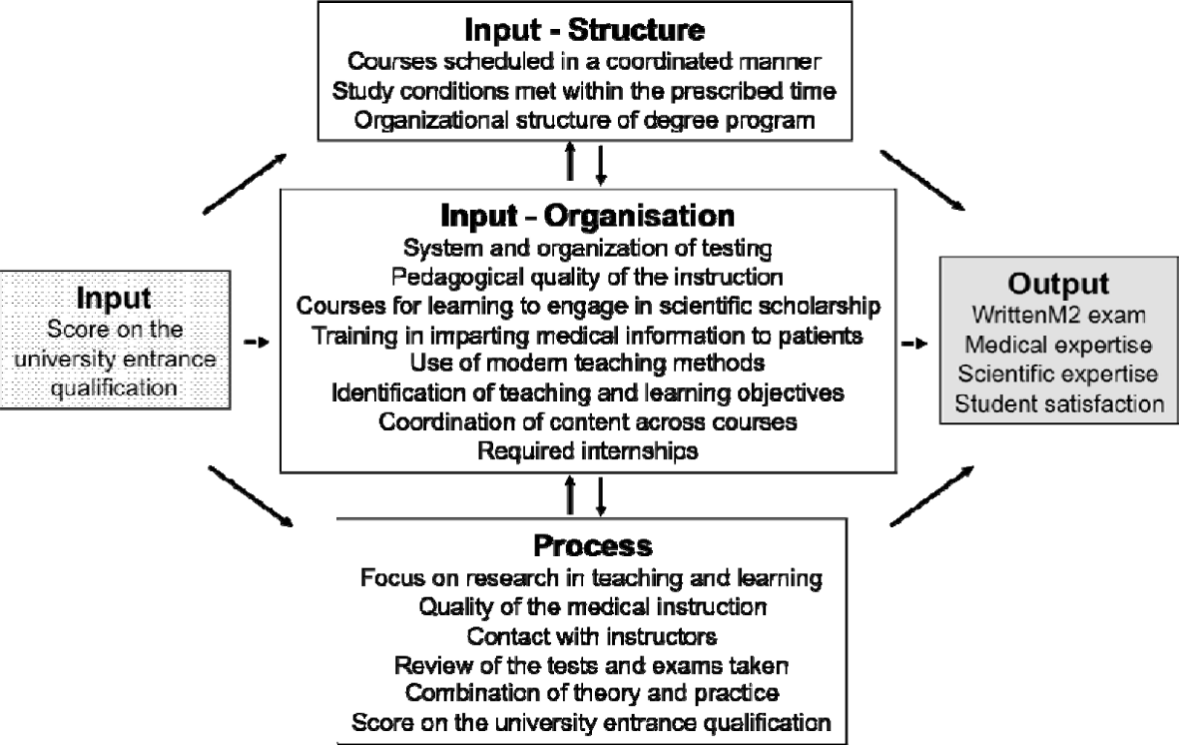
Illustration of the adapted model based on the analytical model for graduate surveys.

In Schomburg’s analytical model (see Figure 1 (Fig. 1)) study success *(output)* is influenced by university-specific aspects that he subdivides into two areas: *input* (study program and study conditions) and process (teaching, learning). Due to its complexity,* input* represents a too general a unit of analysis for posing specific questions about quality assurance. Moreover, delineating clearly between *input* and process is often difficult. Thus, subdividing this area is recommended (see Figure 2 (Fig. 2)). On a macro-level, higher-level curricular aspects arising from the program’s structure become effective and can be steered by the administrative bodies of the medical schools and universities and by higher-level mandates, for instance the German medical licensing regulations *(input/structure)*.

In contrast, at the micro-level many organizational aspects can exert influence on teaching quality *(input/teaching organization)*. Belonging to this are personnel development measures, such as medical teaching programs or implementing a catalogue of learning objectives. These aspects can be affected by both the medical school and the instructors. The area* teaching process* includes factors that primarily arise during the teaching-learning process between instructors and students. The influence of these three areas on study success is explored in this study.

## Aim

The aim of this study was to analyze potential factors influencing study success regarding the Freiburg medical degree program. Of particular interest were the aspects pertaining to teaching and learning, aspects over which the medical school can exert direct influence.

## Method

### Samples

This study draws on data from three graduate surveys of the graduating classes for 2007-2008, 2008-2009, and 2009-2010 from the Freiburg Medical Faculty. All graduates were enrolled at a time when the 2002 German medical licensing regulations, ÄAppO [12], [13], were being implemented. The surveys were conducted as part of the collaborative project, *Study Conditions and Professional Success – KOAB*, led by the International Center for Higher Education Research (INCHER) in Kassel. The surveys took place one and a half to two years after graduation. The gross response rate was calculated based on the overall sample size without deducting any missing data, for instance as a result of unknown addresses. The completion rate was between 46 and 55 percent for the three graduating classes. Sample statistics are presented in Table 1 (Tab. 1). The samples are representative in regard to the parameters of sex, age, final grade and score on the university entrance qualification; however, they are not in reference to citizenship. Graduates holding foreign citizenship are under-represented in the samples.

#### Study success indicators

Study success was operationalized using four indicators. Of the scores given for the M2 exam, only the score for the written part was included, since the objectivity, reliability and validity of oral assessments are often questioned [14]. The other indicators for study success were student satisfaction, as assessed with one item (1=highly satisfied, 5=highly dissatisfied), self-assessed medical expertise (CR-α_JG08-10_=.86) and self-assessed scientific expertise (CR-α_JG08-10_=.66), both measured using the scales of the Freiburg Questionnaire to Assess Competencies in Medicine (FKM) [15]. The FKM is integrated in the graduate surveys, its items having five-point Likert scales ranging from 1 *very much so *to 5 *not at all*.

The four study success indicators hardly or only moderately correlate with each other. The correlation coefficients fall between r=.02 and r=.52.

#### Selected predictor variables

Sixteen items addressing study conditions and acquisition of competencies were taken from the questionnaire,* Fragebogen für medizinische Studiengänge*, used in the KOAB project. The study authors (S.B. and M.G.) identified the items which were particularly relevant or of special interest to the study of medicine. A version of the survey can be found at http://koab.uni-kassel.de/images/download/jg10w1_fb_spez_medizin.pdf.

The selected items were then assigned by the same authors to the following three areas of influence in the modified model (see Figure 2 (Fig. 2)): Input/Structure (3 items), Input/Organization (8 items) and Teaching/Process (5 items) (see Figure 2 (Fig. 2)). Response to these items was possible using a five-point Likert scale (1=*very good*, 5=*very poor*).

Figure 2 (Fig. 2) illustrates how Schomburg’s model was modified for this study. The potential factors influencing study success scrutinized here are represented in the modified model. Assuming that the score on the university entrance qualification represents a highly influential and individual characteristic, this variable was assigned to each area as control variable.

#### Statistical evaluation

Multiple regression analyses based on all three (predictor) areas were carried out for each of the four indicators of study success (written M2 exam score, medical expertise, scientific expertise, and student satisfaction) [16]. They were undertaken stepwise to identify which predictors could explain the largest percent of each study success indicator. To ascertain the degree to which the results are stable, the analyses were carried out using the data from the three samples.

## Results

An overview of the results is presented in Table 2 (Tab. 2).

### Written M2 exam

With the models calculated using the predictors from Input/Structure, up to 18 percent of the variance for the M2 exam criterion could be explained. In all three samples, the predictors *fulfillment of program requirements within the time intended* and the control variable* score on the university entrance qualification* demonstrate significant and thus important beta weights for predicting the written M2 score.

Between 8 and 22 percent of the variance can be explained with the variables from Input/Teaching Organization. The only predictor showing significant beta weights in all three samples is the control variable, *score on the university entrance qualification*. This also applies to the results that were seen for Teaching/Process, for which the percentage of the explained variance is between 9 and 15 percent.

#### Medical expertise

With the models determined using the predictors for Input/Structure, between 5 and 16 percent of the variance for the criterion medical expertise could be explained. The variable *structure and curricular sequencing of the degree program* revealed itself to be the most important predictor, with significant beta weights for all three samples.

With the predictors for Input/Teaching Organization, the models for the three samples are able to explain between 4 and 21 percent of the variance. However, there is no predictor with significant beta weights in any of the three samples. The predictors for Teaching/Process could only explain 11 to 32 percent of the variance seen for the criterion medical expertise. In all three samples the variable *combination of theory and practice* had significant beta weights.

#### Scientific expertise

Using the predictors for the three areas, models were calculated with which between 2 and 17 percent of the variance for the criterion scientific expertise could be explained. For Input/Structure, the multiple correlation was significant for only one sample. In the other analyses carried out with the predictors for the two other areas, the multiple correlations were indeed significant, but it was not possible to identify any one predictor that exerted a relevant influence in all three samples.

#### Student satisfaction

With the predictors in the area Input/Structure, 26 to 35 percent of the variance for the criterion student satisfaction can be explained. In all three samples the predictor *structure and curricular sequencing of the degree program* showed significant beta weights.

With the predictors for the area Input/Teaching Organization, between 19 and 33 percent of the variance for the criterion satisfaction with the degree program could be explained. Here also, there is a predictor showing significant beta weights in all three samples. This predictor is *system and organization of testing*. With the predictors in the area Teaching/Process, between 12 and 25 percent of the variance seen for the criterion student satisfaction could be explained. The predictor showing significant beta weights in all three samples is the *combination of theory and practice*.

In summary, models can be identified using the three predictor groups for three of the four study success parameters. These models offer slight to moderate assistance when predicting study success. The explanation of variance is highest for the study parameter *student satisfaction*.

## Discussion

This study explored the extent to which study success in medicine can be predicted and explained in terms of study conditions and programs, both aspects that can be influenced by medical schools. Differentiation was made between program structure, organization of teaching, and the process of teaching. For each of the analyses, the *score on the university entrance qualification* was also taken into account as an important individual background characteristic, since it is known to have strong predictive value for certain aspects of study success [17]. To ensure the validity of the results, the analyses were carried out on data from three graduate surveys. According to a general rule, a minimum of 10 observations are needed per model parameter to achieve a reasonably stable model [18]. The sizes of the three samples were adequate for the investigation undertaken by this study.

In respect to the M2 exam study success indicator, the control variable for the *score on the university entrance qualification* was calculated as an important predictor in the mostly one-step models for the three areas covering teaching conditions. These models explain between 8 and 22 percent of the total variance in the graduate classes surveyed. Individual cognitive abilities (expressed as the score on the secondary school leaving exam [German *Abitur*]) show themselves to be important to study success when compared to teaching conditions, even if their predictive power is rather weak overall. This is most likely explained by the fact that coordination between local curricula and the written state examination is only limitedly possible. To a very great extent, the centralized drafting and organization of the crucial written medical exams (state medical exams) deprive medical schools of an important tool for guiding and steering students’ learning behavior. Students prepare themselves in a very targeted manner not only in regard to test content, but also test format (multiple-choice questions). Since the written M2 exam exclusively measures cognitive ability (primarily knowledge reproduction), it is not surprising that the final grade attained at the secondary school level, which also refers to cognitive ability, is the only consistent predictor for the exam score. In contrast, the local curricula at the medical schools are supposed to enable students to solve problems in practical settings, particularly during the second phase of study covering clinical competencies for which practical skills, approaches and mindsets are necessary. In terms of content there are substantial discrepancies between the curriculum and the state medical examination.

The idea that a discrepancy exists between knowledge-based and practice-based learning is supported by a study by Raupach [19], in which it was possible to measure only a very slight knowledge gain during the practice-based fifth year of study using questions from a US medical exam (USMLE Step 2). The students’ learning behavior while preparing for the M2 exam can also be understood as a reaction to this discrepancy. If they are asked how they are preparing to take the M2 written exam, they will frequently list old exams, specific workbooks, and a 100-day study plan to strategically organize their learning efforts. Materials that they have received or worked on during their time at medical school appear to play a lesser role in comparison.

Finally, the lack of power seen in the variables within the area Input/Organization for predicting the written M2 exam score can also be explained in that these variables are not easy to influence through curriculum.

For the other study success indicators under investigation, the *score on the university entrance qualification* proved to be of little importance as a predictor variable.

In respect to scientific expertise, no variable could be identified that contributed significantly to the prediction of this study success indicator. Either no significant models were identified, or the models only offered minimal explanation of the variance (between 2 and 17 percent). The poor explanatory power for this study success indicator in regard to teaching is possibly an expression of the fact that, contrary to our assumption, this competency area was only minimally and, in these cases, often not integrated into the curriculum in a structured manner at the time the students were enrolled in their studies. It is only very recently that scientific expertise has received increased attention in teaching [20]. It is also conceivable that different predictors must be chosen for scientific expertise than for the practical clinical phase of study.

To predict the study success indicator for medical expertise, the predictors* structure and curricular sequencing of the degree program* and *combination of theory and practice* revealed themselves to be significant in all three samples. This result is not surprising since clarity and transparency of the curricular structure represent an important pre-requisite for teaching quality [21], and an adequate combination of theory and practice is necessary for imparting medical expertise [22], [23]]. It is conspicuous that the predictors in the area Input/Teaching Organization appear to have no importance for imparting medical expertise. This could result from the selection of predictors, or it could be that, although the surveyed graduates were enrolled at the university at the time when the new licensing regulations were implemented, the conceptual changes (teacher training and qualification, use of modern learning forms, etc.) took place successively over a longer period of time so that those surveyed did not necessary experience the effects of these changes.

In all three samples there were three important predictors for student satisfaction: *structure and curricular sequencing of the degree program, system* *and organization of testing* and *combination of theory and practice*. Two of these three predictors are also relevant for predicting medical expertise. A correlation between the study success indicators for medical expertise and student satisfaction is not surprising, since it can be presumed that those who have successfully completed a medical degree program during which they have perceived themselves as professionally competent are also satisfied with their studies. In addition, student satisfaction could also be directly influenced by these teaching-related parameters, for instance because a well organized degree program directly coincides with a high level of student satisfaction.

This study leads to the tentative suggestion that medical schools can exert influence on study success (self-assessed medical expertise and student satisfaction) by developing and influencing the teaching-related parameters *structure and curricular sequencing of the degree program* and *combination of theory and practice*. Further studies would be needed to replicate the results of this one.

When considered as a whole, the results suggest that the credit due to medical schools for the study success of their students can only be measured with difficulty when using a testing instrument based virtually on knowledge alone, such as the written M2 exam. It was, however, possible to determine slight, yet significant effects on the self-assessed level of medical expertise. In order to verify this self-assessment, the results of the oral practical section of the M2 (now M3) exam should be drawn upon instead or the results of other competency-based testing formats. However, these must meet the requisite quality criteria (objectivity, reliability, validity) [24]. This is usually not the case and thus the explanatory power of these scores is limited [14], [25].

The differences in the models regarding the study success indicators for study success in the analyses of the three areas could arise from the fact that the students who graduated between 2007 and 2010 studied under a *shifting curriculum* since they enrolled after the amended licensing regulations of 2002 came into effect. The revised regulations led to many curricular changes that were successively implemented over many years, a process that is even now not yet complete. At the time, the second phase of study was being restructured in Freiburg [13] so that, during the course of their studies, the surveyed students experienced curricular changes in different stages of implementation.

## Limitations

That scientific expertise and medical expertise were measured using a self-assessment tool must be acknowledged as a limitation, since biases in these study success indicators cannot be ruled out. Similar limitations may also apply to the other results of the graduate surveys. The influence of social expectancy when responding to the questions cannot be ruled out. Also, a recall bias cannot be excluded since the graduates evaluated their studies approximately one and half years after completion. Furthermore, the potential influencing factors were selected from many variables with which the study conditions were determined. Other variables may also have been suitable. The data in this study have been collected from graduates of the Freiburg Medical School. Further studies would be necessary to establish whether or not these results are also valid in respect to other medical schools.

## Competing interests

The authors declare, that they have no competing interests.

## Figures and Tables

**Table 1:**
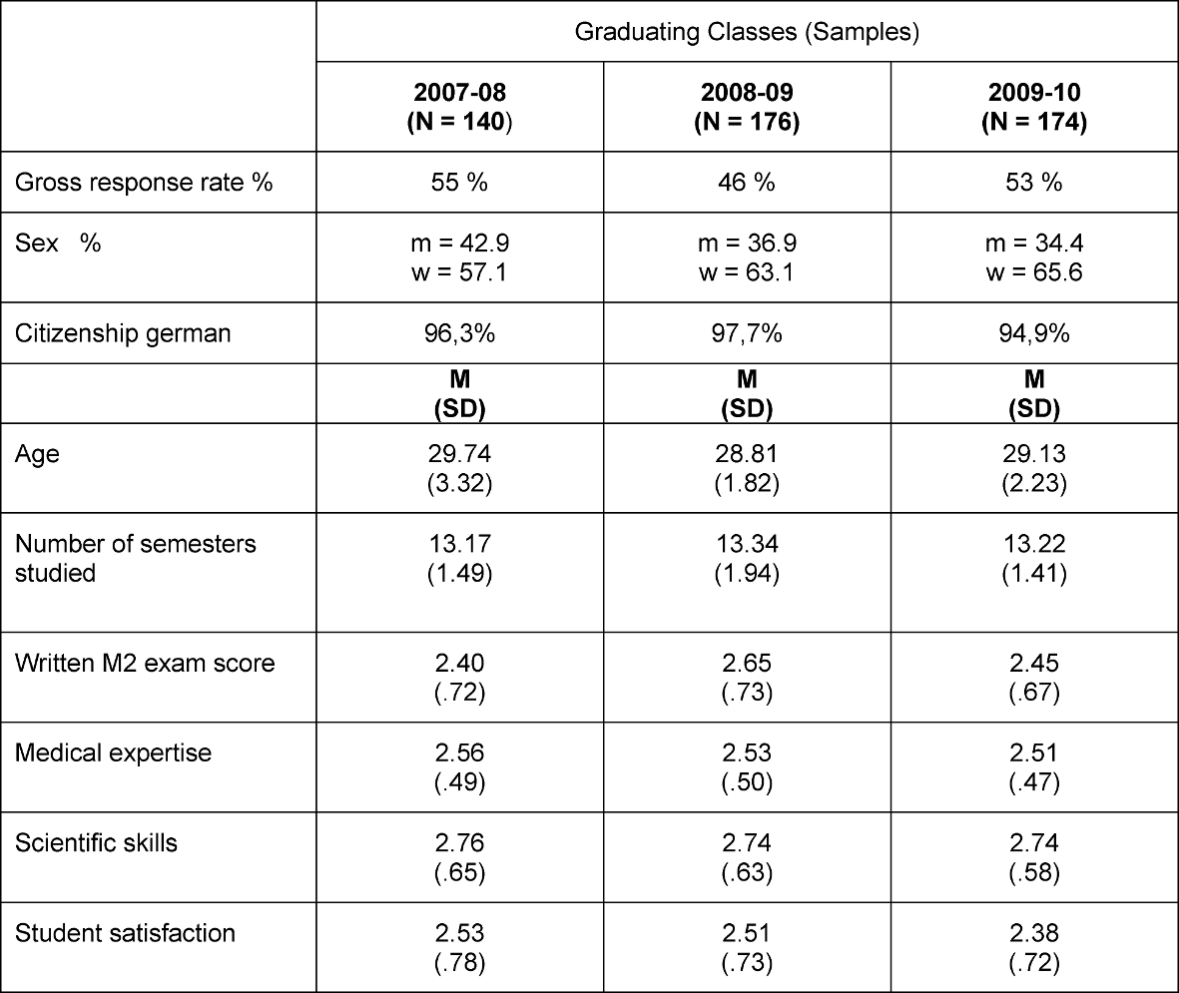
Statistical values of the samples

**Table 2:**
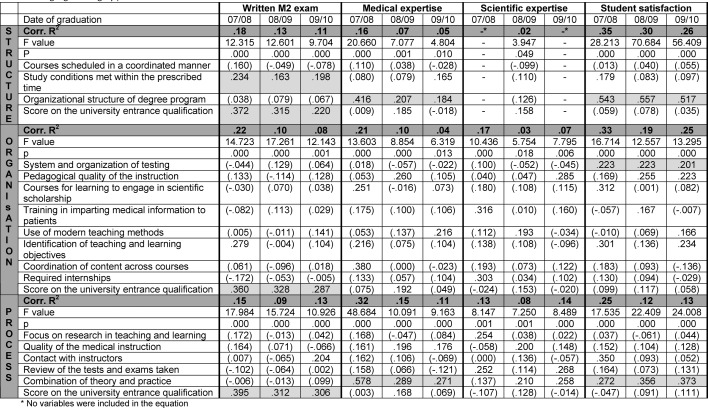
Results of the multiple regression analyses (Insignificant beta weights appear in parentheses. Beta weights for predictors that were significant in all three samples have been highlighted in gray.).
